# Ssu72 attenuates autoimmune arthritis via targeting of STAT3 signaling and Th17 activation

**DOI:** 10.1038/s41598-017-05421-x

**Published:** 2017-07-14

**Authors:** Seung Hoon Lee, Eun-Kyung Kim, Jeong-Eun Kwon, Jin-Kwan Lee, DoHyeong Lee, Se-Young Kim, Hyeon-Beom Seo, Hyun Sik Na, KyoungAh Jung, Seung-Ki Kwok, Chang-Woo Lee, Sung-Hwan Park, Mi-La Cho

**Affiliations:** 1 The Rheumatism Research Center, Catholic Research Institute of Medical Science, College of Medicine, The Catholic University of Korea, Seoul, South Korea; 2 Department of Health Sciences and Technology, SAIHST, Sungkyunkwan University, Seoul, 06351 Korea; 3 Department of Molecular Cell Biology, Sungkyunkwan University School of Medicine, Suwon, 16419 Korea; 4 Impact Biotech, Seoul, 137-040 South Korea; 5 Division of Rheumatology, Department of Internal Medicine, Seoul St. Mary’s Hospital, College of Medicine, The Catholic University of Korea, Seoul, 137-701 South Korea; 6 Laboratory of Immune Network, Conversant Research Consortium in Immunologic Disease, College of Medicine, The Catholic University of Korea, Seoul, South Korea; 7 The Institute for Aging and Metabolic Diseases, College of Medicine, The Catholic University of Korea, Seoul, 06591 South Korea

## Abstract

Signal transducer and activator of transcription 3 (STAT3) orchestrates the differentiation of several cell types, including interleukin-17 (IL-17)-releasing Th17 cells. Dysregulation of Th17 cells results in chronic inflammatory responses. Ssu72 is a C-terminal domain phosphatase required for transcriptional regulation. However, the mechanism by which Ssu72 affects STAT3 activation and Th17 cell differentiation is unclear. Here, we found that Ssu72 overexpression suppresses STAT3 activation and Th17 cell responses *in vitro*. A systemic infusion of Ssu72 attenuates experimental autoimmune arthritis by reducing STAT3 activity and the differentiation of Th17 cells. It also reduces joint destruction, serum immunoglobulin concentrations and osteoclastogenesis but increases the number of marginal zone B cells and B10 cells. These effects are associated with reduced p-STAT3 levels and the suppression of Th17 cell formation *in vivo*. Based on these data, Ssu72 is related to STAT3 activation and the inflammatory response; and Ssu72 overexpression in T-cell-mediated immunity has potential utility for the treatment of autoimmune arthritis.

## Introduction

Autoimmune disorders involve abnormal immune inflammatory responses that lead to improper assaults on healthy tissue, resulting in tissue destruction. Autoimmune disorders are mediated by T cells, which induce chronic and excessive inflammation^1, 2^. CD4^+^ T cells differentiate into either effector T cells or regulatory T (Treg) cells; these CD4^+^ T cell lineages regulate the immune responses that contribute to the pathogenesis of autoimmune diseases^3^.

Rheumatoid arthritis (RA) is a complex systemic inflammatory autoimmune disease that causes excessive and chronic inflammation in joints, destruction of the adjacent cartilage, and disability. T cells play a crucial role in the pathogenesis of RA by stimulating inflammatory cell infiltration into the inflamed synovium, in which CD4^+^ T cells maintain rheumatoid inflammation^4^. Interleukin-17 (IL-17)-secreting CD4^+^ T (Th17) cells cause auto-inflammation^5^, whereas Treg cells produce immunosuppressive cytokines, such as IL-10, that limit the immune response in RA pathogenesis^6^. Recently, the reciprocal regulation mediated by Th17 and Treg cells has been suggested as a therapeutic strategy for treating RA^7^.

Signal transducer and activator of transcription 3 (STAT3), a DNA-binding molecule, plays an essential role in the production of several cytokines. STAT3 is recognized as a critical player in the development of autoimmune diseases^8^. STAT3 activation induces Th17 cell differentiation through upregulation of *Il17a* gene expression^9^. Based on accumulating evidence, STAT3 inhibition may be a potential target for RA therapy because it ameliorates the severity of experimental autoimmune arthritis and helps to regulate the balance between Th17 and Treg cells^7^.

Ssu72 is a C-terminal domain (CTD) phosphatase with an indispensable role in transcription. Ssu72 plays an essential role in mRNA biogenesis by interacting with transcription factors^10, 11^. The Ssu72 structure resembles the core fold of protein tyrosine phosphatases, and Ssu72 exhibits phosphatase activity^12–14^.

We hypothesized that Ssu72 suppresses STAT3 activation and is a critical and highly conserved protein involved in autoimmune diseases. A prospective study was undertaken to characterize the biochemical activity of Ssu72 in the immune response. We performed both *in vivo* and *in vitro* experiments to identify the mechanisms underlying Ssu72 overexpression during RA development and the consequences of its overexpression. First, we assessed the anti-inflammatory activities of Ssu72 and its ability to inhibit STAT3. Second, we investigated whether Ssu72 overexpression ameliorated RA using an *in vivo* mouse model. Finally, we evaluated the effects of Ssu72 on the balance between Th17 and Treg cells in relation to the STAT3 pathway in a mouse model of RA to identify the mechanism by which Ssu72 and STAT3 impair inflammation.

## Results

### Ssu72 overexpression reduces STAT3 activation *in vitro*

**Figure 1:**
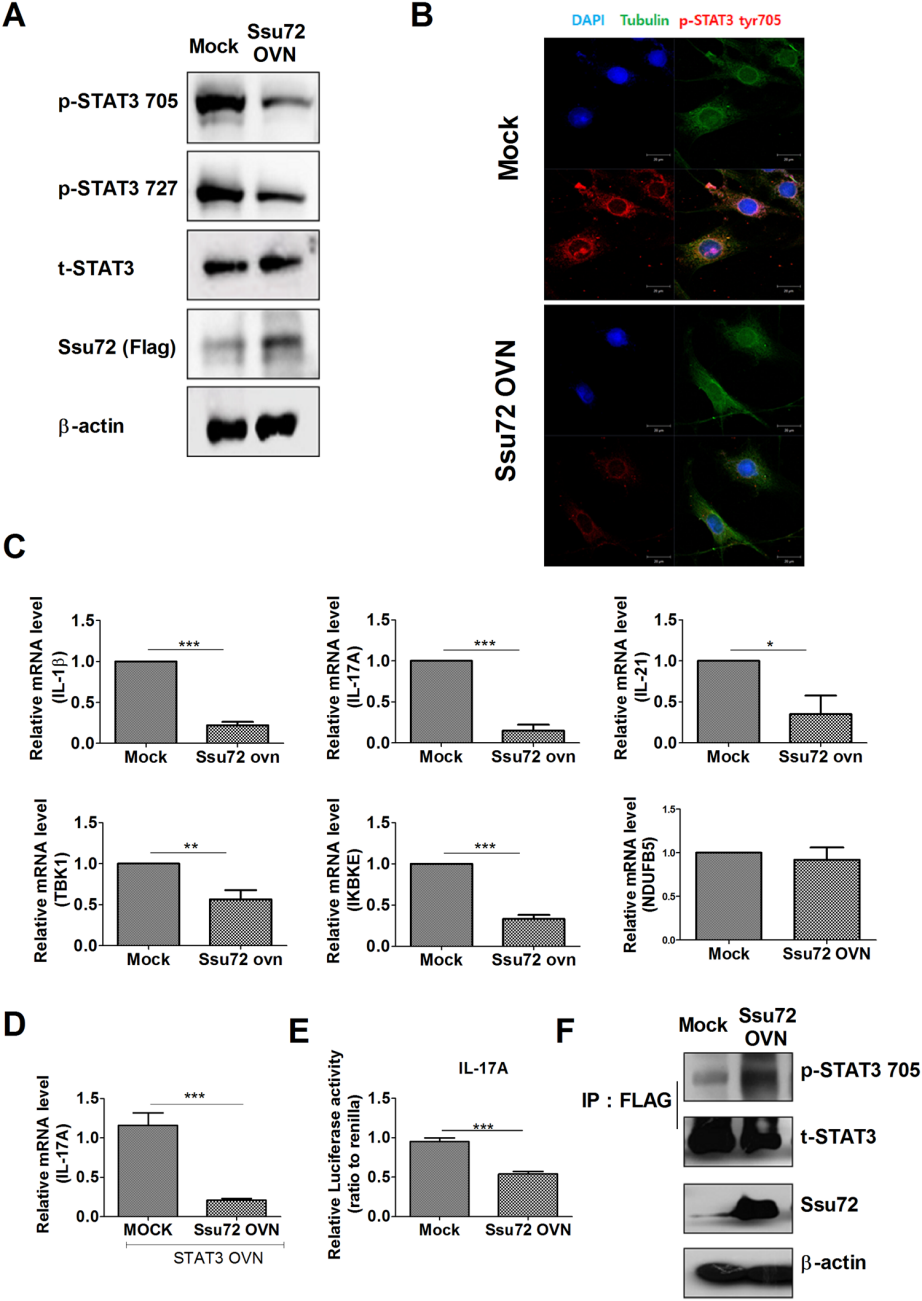
Ssu72 regulates STAT3 activation. (**A**) NIH-3T3 cells were transfected with either the mock or Ssu72 overexpression vector and stimulated with IL-6 (20 ng/ml) for 1 h. Cells were used to detect the p-STAT3 Tyr705 and p-STAT3 Ser727 levels. (**B**) NIH-3T3 cells were stained with DAPI (blue) and antibodies against tubulin (green) and p-STAT3 Tyr705 (red). Confocal scanning microscopy was performed to detect the p-STAT3 Tyr705 and p-STAT3 Ser727 levels in the transfected cells. (**C**) NIH-3T3 cells were transfected with the expression vector and stimulated with IL-6 (20 ng/ml) for 1 h. (**D**) Cells were transfected with STAT3 overexpression vectors and either the mock or Ssu72 overexpression vector. The expression levels of the *Il17a* mRNA were measured using real-time PCR. (**E**) NIH-3T3 cells were transfected with the *Il17a* promoter construct and either mock or Ssu72 expression vectors. Luciferase activity was then detected. (**F**) Lysates from the transfected NIH-3T3 cells were immunoprecipitated with the anti-FLAG antibody and immunoblotted with anti-p-STAT3 Tyr705, anti-p-STAT3, and anti-Ssu72 antibodies. The data represent the mean ± SD from three independent experiments. Statistical analyses were conducted using the nonparametric Mann-Whitney *U*-test (*P < 0.05, ***P < 0.01).

Because Ssu72 exhibits phosphatase activity^13, 14^, we overexpressed Ssu72 in NIH-3T3 cells by transfecting them with an *Ssu72* overexpression vector. Then, cells were stimulated with IL-6 and the level of phosphorylated STAT3 (p-STAT3) was measured. Ssu72 overexpression reduced the levels of p-STAT3 Tyr705 and Ser727 in NIH-3T3 cells (Fig. 1A). We also detected the p-STAT Tyr705 levels in the cells using confocal scanning microscopy (Fig. 1B). Expression of the catalytic mutant of the Ssu72 phosphatase (C12S) increased the p-STAT Tyr705 levels in NIH-3T3 cells (Supplementary Figure 1A). Ssu72 overexpression decreased STAT3-dependent luciferase activity, but the Ssu72 (C12S) mutant upregulated the luciferase activity of the *Il17a* promoter in the same cells (Supplementary Figure 1B). Ssu72 overexpression significantly reduced the mRNA levels of inflammatory cytokines, including *Il17a*, in NIH-3T3 cells. Ssu72 overexpression also significantly reduced the levels of the *TBK1* and *IKBKE* mRNAs. But, mRNA expression of *NDUFB5* which is a STAT3-independent gene was not affected by Ssu72 overexpression (Fig. 1C). Moreover, the levels of the *Il17a* mRNA were also decreased by Ssu72 overexpression in *STAT3*-overexpressing NIH-3T3 cells (Fig. 1D). According to the analysis of the *Il17a* promoter using a luciferase reporter system, Ssu72 overexpression reduced the luciferase activity of the *IL17a* promoter (Fig. 1E). Ssu72 bound directly to STAT3 (Fig. 1F). STAT3 activation induces inflammation by promoting proinflammatory cytokine production^15^. Thus, Ssu72 may downregulate STAT3 activation and reduce inflammation *in vitro*.

### Downregulation of Ssu72 increases inflammatory responses *in vitro*

**Figure 2:**
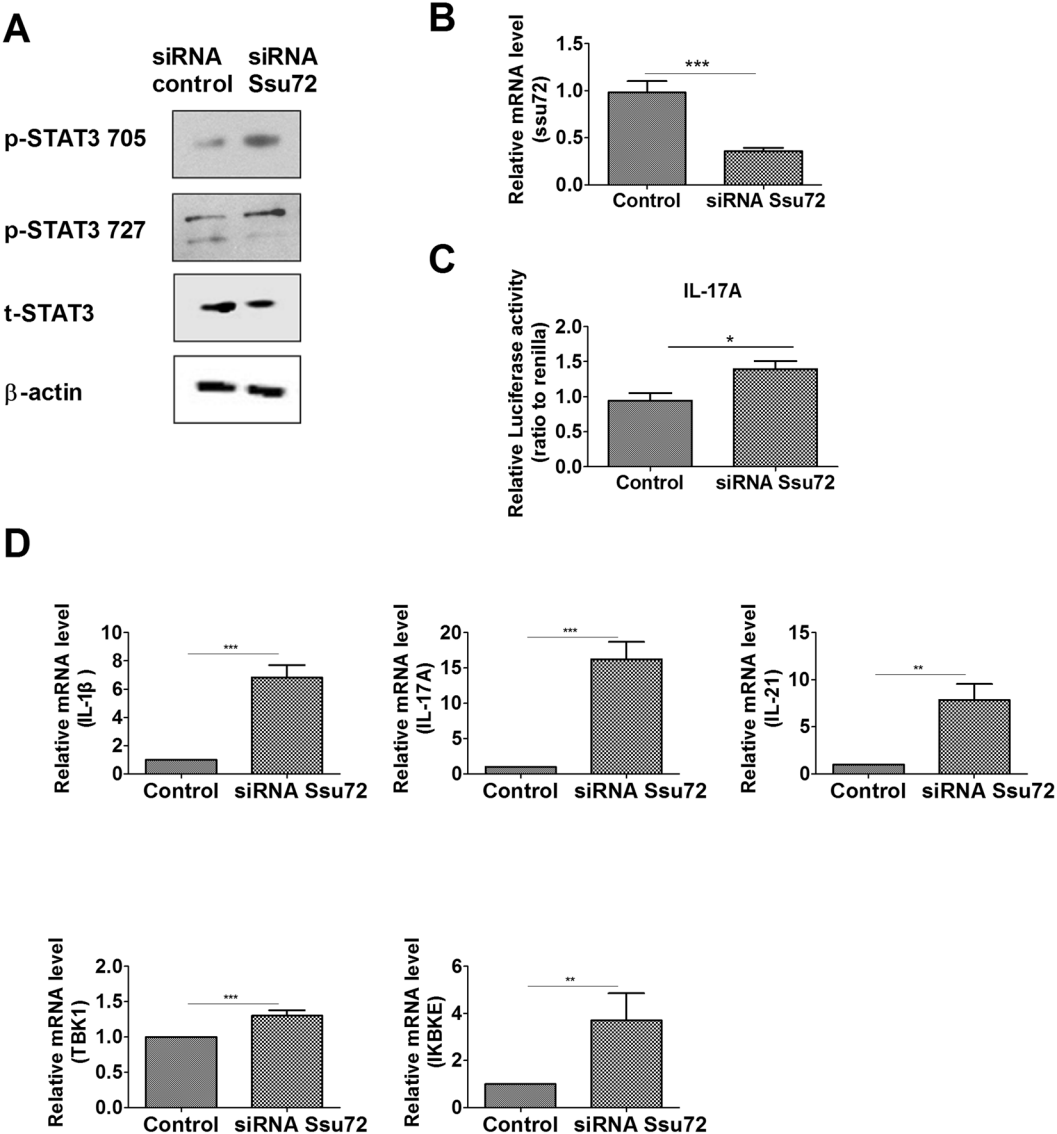
Ssu72 controls inflammatory responses *in vitro*. (**A**) NIH-3T3 cells were transfected with a control siRNA or Ssu72 siRNA and stimulated with IL-6 (20 ng/ml) for 0.5 h. Cells were used to examine the p-STAT3 Tyr705 and p-STAT3 Ser727 levels. (**B**) Expression levels of the *Ssu72* mRNA in cells transfected with the siRNAs were measured by real-time PCR. (**C**) NIH-3T3 cells were transfected with the *Il17a* promoter construct and either the siRNA control or siRNA Ssu72 to detect luciferase activity. (**D**) NIH-3T3 cells were transfected with siRNAs and stimulated with IL-6 (20 ng/ml) for 0.5 h. Real-time PCR was performed to measure the expression levels of the *IL-1β*, *IL-17A*, *IL-21*, *TBK1*, and *IKBKE* mRNAs. The data represent the mean ± SD from three independent experiments. Statistical analyses were conducted using the nonparametric Mann-Whitney *U*-test (*P < 0.05, **P < 0.03, ***P < 0.01).

In contrast, reducing *Ssu72* expression with a siRNA resulted in increased p-STAT3 Tyr795 and Ser727 levels in the transfected cells (Fig. 2A and B). Downregulation of Ssu72 significantly increased the luciferase activity of the *Il17a* promoter in the transfected cells (Fig. 2C). Moreover, the mRNA levels of these inflammatory mediators were significantly increased in the cells transfected with the Ssu72 siRNA (Fig. 2D). STAT3 controls inhibitor of kappa light polypeptide gene enhancer in B cells, kinase epsilon (IKBKE) production^16^. Additionally, TANK binding kinase 1 (TBK1) and IKBKE, two members of the IκB kinase family, mediate the inflammatory response^17, 18^. Based on these findings, Ssu72 may regulate the inflammatory response by binding to STAT3.

### Ssu72 overexpression exerts a therapeutic effect on the mouse model of collagen-induced arthritis (CIA) ***in vivo***

**Figure 3:**
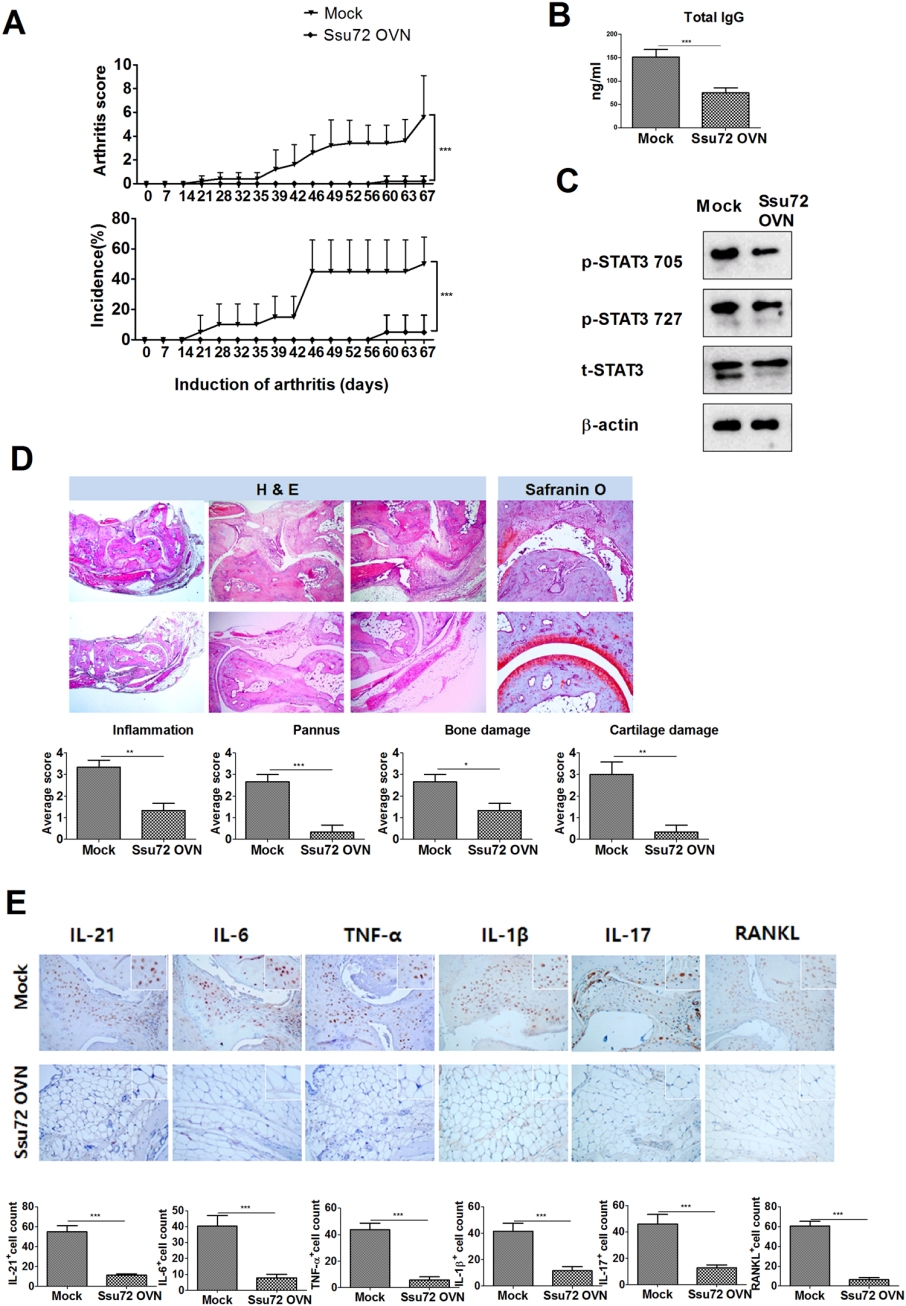
Ssu72 ameliorates the development of CIA. (**A**) The Ssu72 overexpression or mock vector was administered systemically to mice with CIA once per week. The clinical scores and incidence of CIA were measured in these mice (***P < 0.01, n = 10). (**B**) Total IgG levels were measured in each group (***P < 0.01, n = 10). (**C**) The levels of p-STAT3 Tyr705 and p-STAT3 Ser727 in splenocytes from mice with CIA (mock or Ssu72-overexpressing) that had been stimulated with IL-6 (20 ng/ml) for 1 h were examined by western blot analysis. (**D**) Joint tissues from mice with CIA were stained with hematoxylin and eosin (original magnification, 40× or 200×, n = 6) and Safranin O (original magnification, 40×, n = 6). (**E**) Immunohistochemical staining was used to detect IL-6, IL-21, IL-17, IL-1β, TNF-α and RANKL in the synovium of mice with CIA (mock or Ssu72-overexpressing; (original magnification, 200× or 400×, n = 6). The data represent the mean ± SD from three independent experiments. Statistical analyses were conducted using the nonparametric Mann-Whitney *U*-test (*P < 0.05, **P < 0.03, ***P < 0.01).

Mice were intravenously injected with either the Ssu72 overexpression vector or a control mock vector 1 week after collagen type II (CII) immunization (Supplementary Figure 2). Administration of the Ssu72 overexpression vector attenuated the severity of arthritis, as indicated by the decrease in the mean arthritis score and amelioration of arthritic tissue pathology in the affected joints, as revealed by histological approaches (Fig. 3A). The total IgG levels were significantly lower in the Ssu72-overexpressing group than in the mock group (Fig. 3B). In splenocytes, Ssu72 overexpression reduced the levels of p-STAT3 Tyr705 and Ser727 (Fig. 3C). According to the histological analysis, infiltration by immune cells, joint destruction, and cartilage damage were markedly suppressed following the administration of the Ssu72 overexpression vector (Fig. 3D). The expression of proinflammatory cytokines, such as IL-1β, IL-17A, and tumor necrosis factor-α (TNF-α), was significantly lower in the arthritic joints of the Ssu72-overexpressing group than in the mock group (Fig. 3E). Thus, Ssu72 reduces the severity of CIA by downregulating STAT3 activation and autoantibody production.

### Ssu72 overexpression inhibits osteoclastogenesis ***in vivo*** in the mouse model of CIA

**Figure 4:**
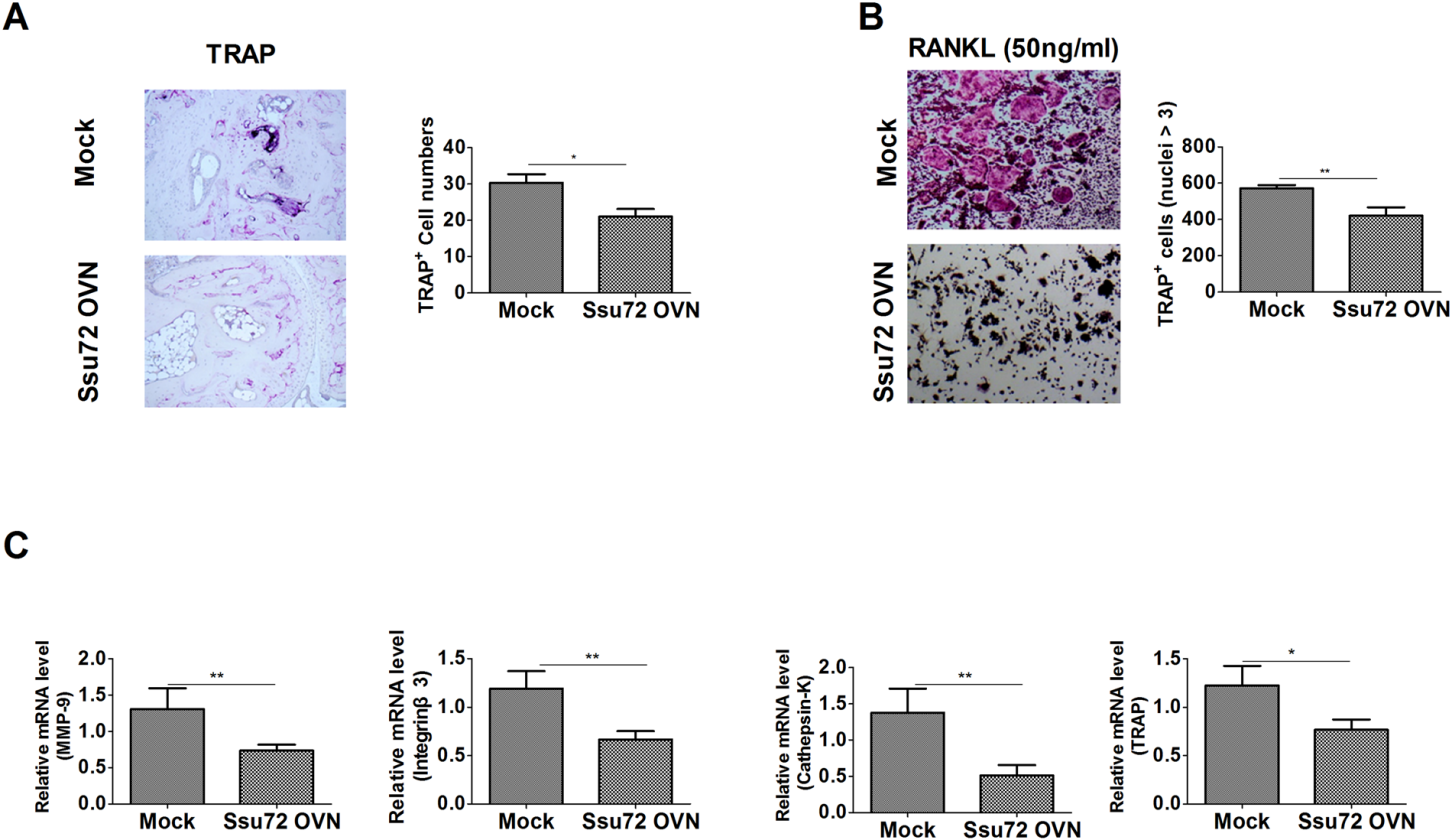
Ssu72 inhibits the progression of osteoclastogenesis. (**A**) TRAP expression in the synovium of mice with CIA (mock or Ssu72-overexpressing) was observed using immunohistochemical staining (original magnification, 200× or 400×, n = 6). (**B** and **C**) Bone marrow cells from mice with CIA (mock or Ssu72-overexpressing) were cultured with macrophage colony-stimulating factor (M-CSF) (10 ng/ml) and RANKL (50 ng/ml). Cells were fixed, stained for TRAP, and the number of TRAP^+^ cells was counted using a light microscope (original magnification 100×, n = 6). Real-time PCR was performed to measure the relative mRNA levels of osteoclastogenic markers (n = 6). The data represent the mean ± SD from three independent experiments. Statistical analyses were conducted using the nonparametric Mann-Whitney *U*-test (*P < 0.05, **P < 0.03, ***P < 0.01).

Tartrate-resistant acid phosphatase (TRAP) expression in arthritic joints was reduced following the administration of the Ssu72 overexpression vector (Fig. 4A). Osteoclastogenesis and the mRNA transcript levels of osteoclastogenesis markers were also significantly lower in the Ssu72-overexpressing group than in the mock group (Fig. 4B and C). Thus, Ssu72 ameliorates CIA by reducing osteoclastogenesis.

### Ssu72 overexpression has therapeutic effects on the mice with CIA by controlling the balance between Th17 and Treg cells ***in vivo***

**Figure 5:**
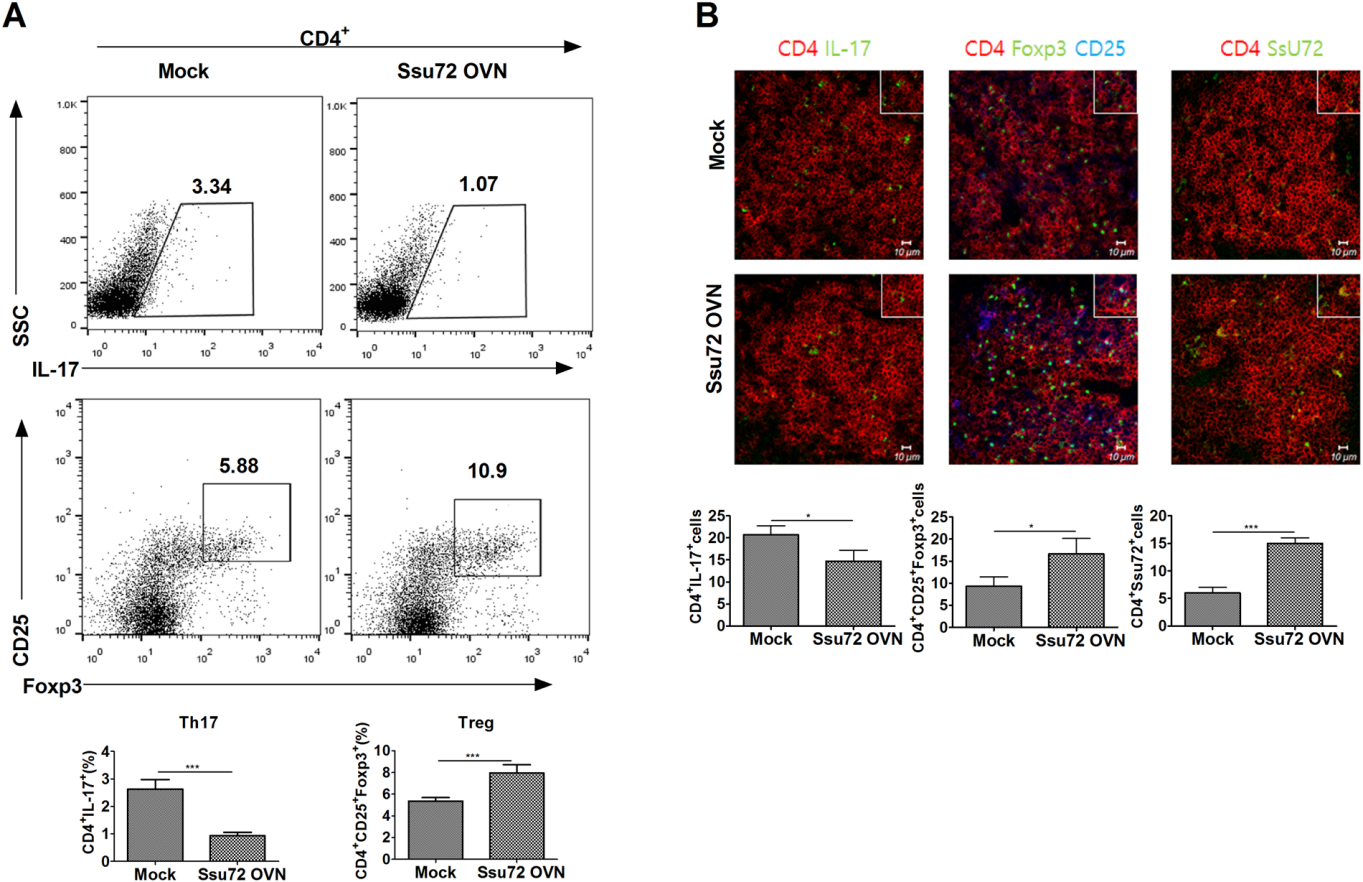
Ssu72 regulates the reciprocal balance between Th17 and Treg cells in mice with CIA. (**A**) Populations of IL-17-, CD25-, and Foxp3-producing CD4^+^ T cells were analyzed by intracellular flow cytometry. (**B**) Spleens from mice with CIA (mock or Ssu72-overexpressing) were subjected to immunostaining to detect the CD4^+^IL-17^+^, CD4^+^CD25^+^FOXP3^+^ and CD4^+^Ssu72^+^ cells (scale bar, 10 μm). Cell numbers were counted in four independent quadrants. The data represent the mean ± SD from three independent experiments. Statistical analyses were conducted using the nonparametric Mann-Whitney *U*-test (*P < 0.05, **P < 0.03, ***P < 0.01, n = 6).

Among mouse splenocytes, Th17 cell differentiation was significantly reduced in the Ssu72-overexpressing group compared with that in the mock group. In contrast, Treg cell differentiation was markedly increased in the Ssu72-overexpressing group compared with that in the mock control group (Fig. 5A). The mRNA levels of genes encoding proinflammatory cytokines, TBK1 and IKBKE were reduced in splenocytes from the Ssu72-overexpressing group (Supplementary Figure 3A and B). The number of CD4^+^ IL-17^+^ cells in the spleen was significantly lower and numbers of CD4^+^CD25^+^ forkhead box P3 (FOXP3)^+^ and CD4^+^SSu72^+^ cells were significantly higher in the Ssu72-overexpression group than in the mock control group (Fig. 5B). Moreover, fewer CD4^+^ cells expressed p-STAT3 Tyr705, p-STAT3 Ser727, TBK1, and IKBKE, and p-STAT5 expression was higher in spleens from the Ssu72-overexpressing group than in the mock control group (Supplementary Figure 4). Th17 cell differentiation was significantly reduced in lymph nodes, whereas Treg cell differentiation was markedly increased in the Ssu72-overexpressing group compared with that in the mock control group (Supplementary Figure 5A). The mRNA levels of proinflammatory cytokines, *TBK1* and *IKBKE* were decreased in lymph nodes of the Ssu72-overexpressing group (Supplementary Figure 5B and C).

### Ssu72 overexpression exerts therapeutic effects on the mice with CIA by controlling the B cell response ***in vivo***

**Figure 6:**
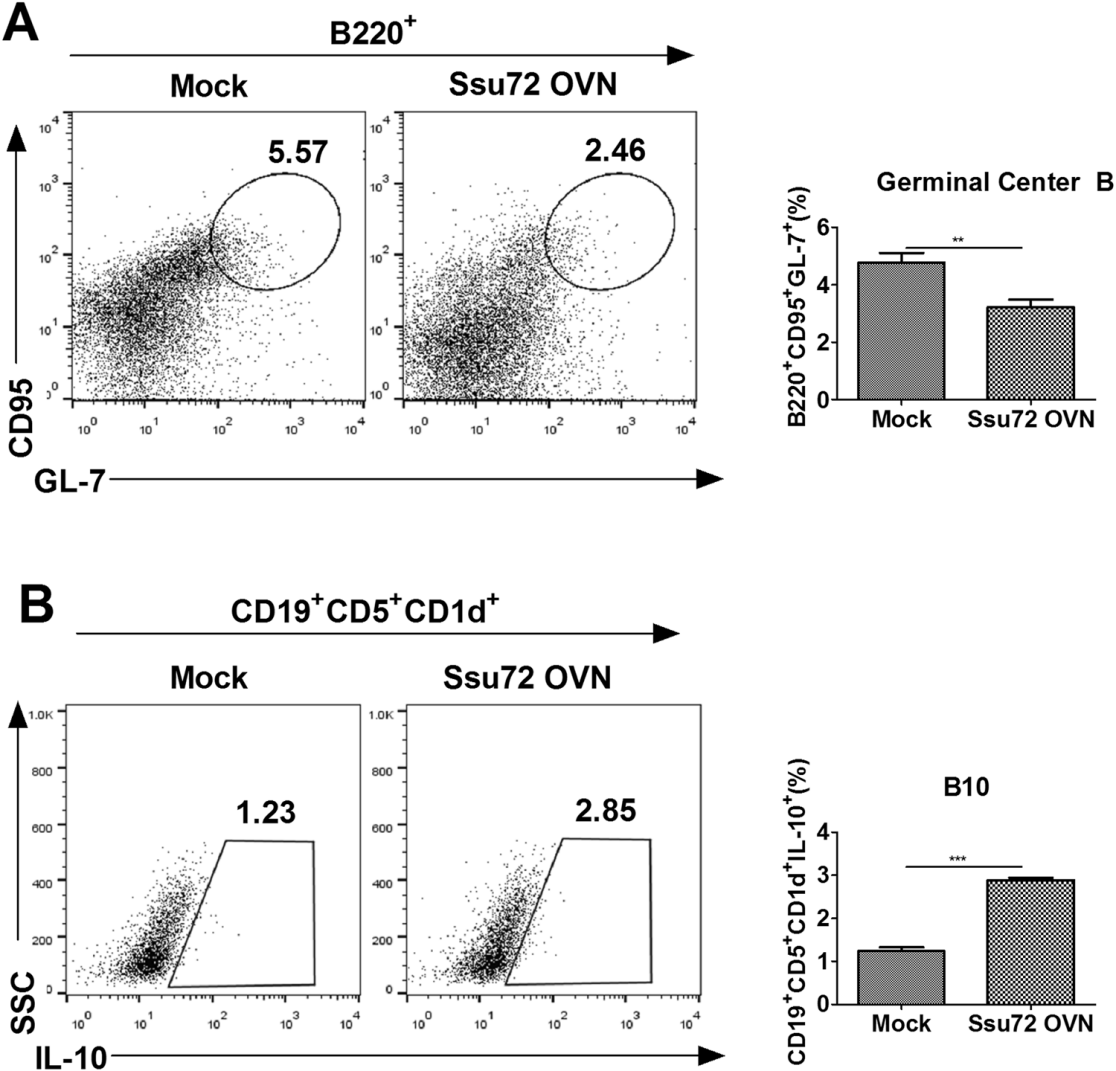
Ssu72 overexpression exerts therapeutic effects on CIA in an *in vivo* model by controlling B cell responses. (**A**) The populations of GL-7-producing B220^+^CD95^+^ B cells in splenocytes from mice with CIA (mock or Ssu72-overexpressing) were analyzed by intracellular flow cytometry. (**B**) Populations of IL-10-producing CD19^+^CD5^+^CD1d^+^ B cells in splenocytes from mice with CIA were analyzed by intracellular flow cytometry. The data represent the mean ± SD from three independent experiments. Statistical analyses were conducted using the nonparametric Mann-Whitney *U*-test (**P < 0.03, ***P < 0.01, n = 6).

Germinal center B cell differentiation is a key process involved in autoantibody production^19^, whereas the differentiation of B10 cells mediates anti-inflammatory responses by inducing IL-10 production^20^. The differentiation of germinal center B cells was significantly lower in the splenocytes of the Ssu72-overexpressing group than in the mock group (Fig. 6A), but not in the lymph nodes (Supplementary Figure 6A). In contrast, B10 cell differentiation was significantly increased in the spleen and lymph nodes of the Ssu72-overexpressing group compared that in with the mock group (Fig. 6B and Supplementary Figure 6B). Based on these findings, Ssu72 ameliorates CIA by promoting B10 cell differentiation and downregulating both autoantibody production and osteoclastogenesis. Thus, Ssu72 may attenuate CIA progression by limiting inflammatory responses and osteoclastogenesis *in vivo*.

### Ssu72 downregulates Th17 cell differentiation and IL-17 level by reducing STAT3 activation ***in vitro***

**Figure 7:**
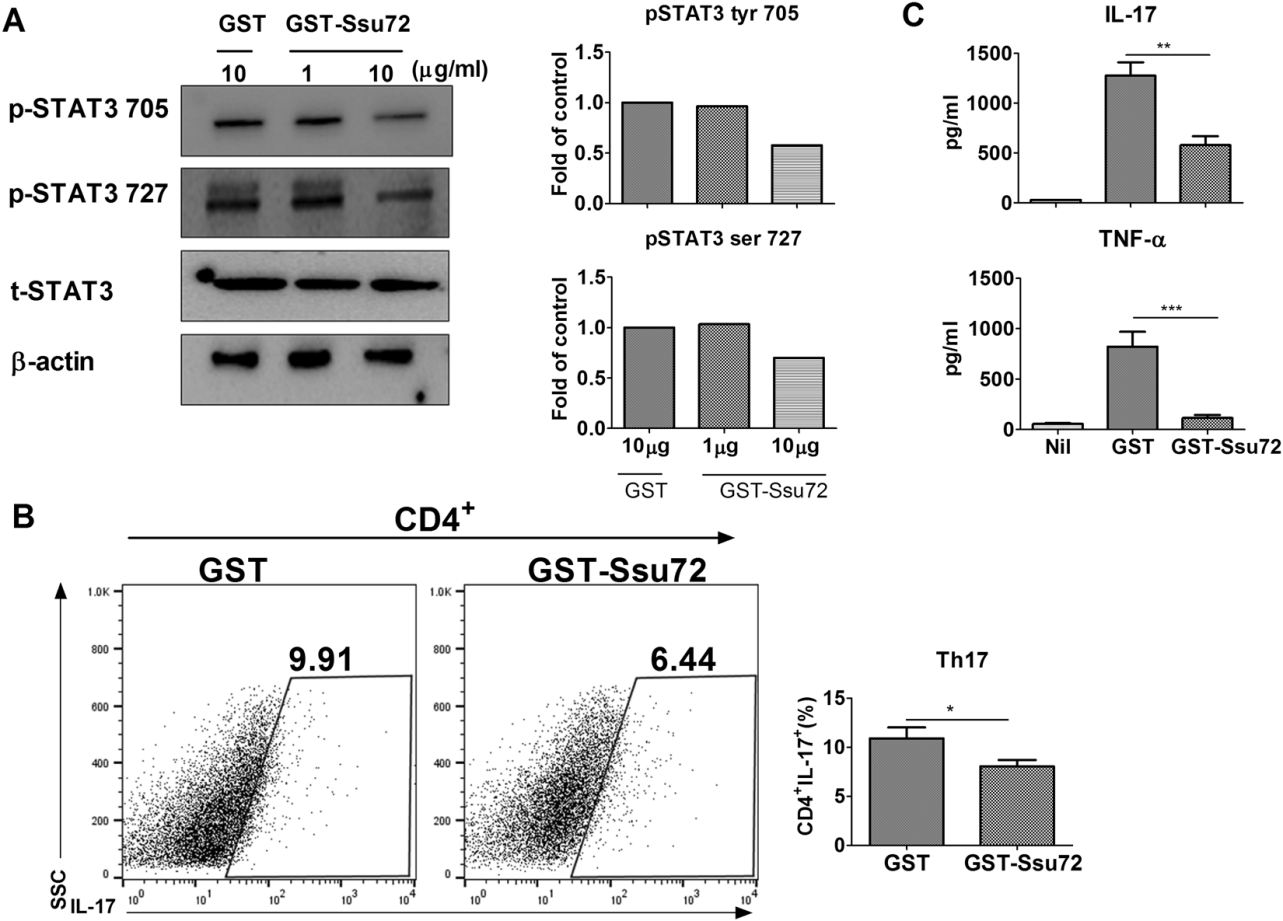
Ssu72 reduces STAT3 activation and Th17 cell differentiation. (**A**) Splenocytes from mice with CIA were stimulated with IL-6 (20 ng/ml) for 1 h and then treated with either GST or GST-Ssu72. (**B**) Splenocytes from mice with CIA were cultured under Th17 conditions for 72 h and then the number of CD4^+^IL-17^+^ cells was quantified. Statistical analyses were conducted using one-way ANOVA with Bonferroni’s post hoc test. (**C**) IL-17 and TNF-α expression in culture supernatants was measured using ELISA. The data represent the mean ± SD from three independent experiments. Statistical analyses were conducted using the nonparametric Mann-Whitney *U*-test (*P < 0.05, **P < 0.03, ***P < 0.01, n = 5).

**Figure 8:**
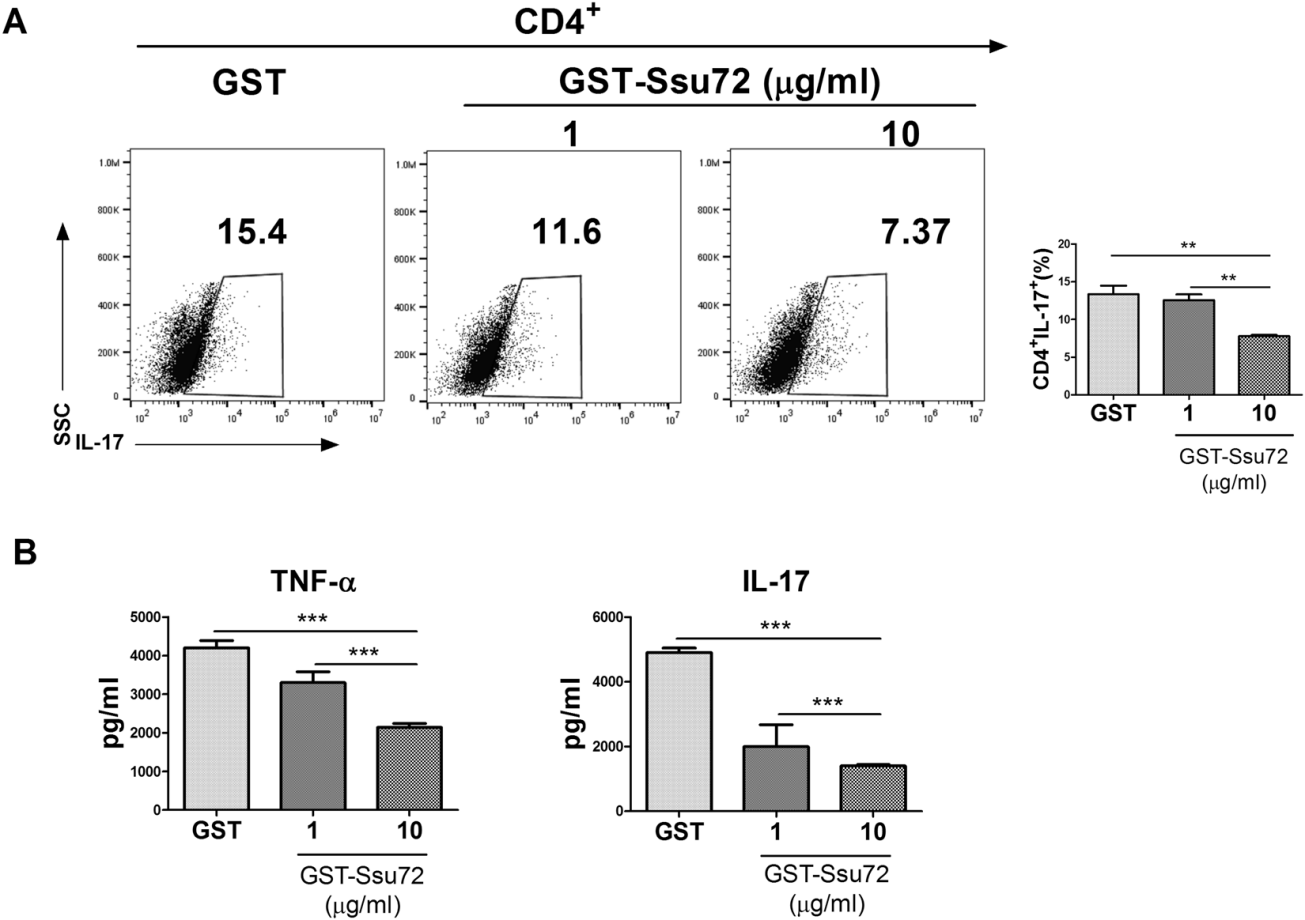
Ssu72 downregulates Th17 cell differentiation and IL-17 level. (**A**) CD4^+^ T cells from CIA mice splenocytes were cultured under Th17 conditions for 72 h and then the number of CD4^+^IL-17^+^ cells was quantified. Statistical analyses were conducted using one-way ANOVA with Bonferroni’s post hoc test. (**C**) Concentration of IL-17 and TNF-α in culture supernatants was measured using ELISA. The data represent the mean ± SD from three independent experiments. Statistical analyses were conducted using the nonparametric Mann-Whitney *U*-test (*P < 0.05, **P < 0.03, ***P < 0.01, n = 5).

We found that glutathione S-transferase (GST)–Ssu72 decreased the expression of p-STAT3 Tyr705 and Ser727 in mice splenoyctes stimulated by IL-6 (Fig. 7A). Moreover, GST-Ssu72 treatment reduced significantly Th17 cell differentiation (Fig. 7B). Significantly lower concentrations of IL-17 and TNF-α were observed in culture supernatants from the cells treated with GST–Ssu72 compared with those from GST treated the cells (Fig. 7C). We isolated CD4^+^ T cells from mice splenoyctes and also observed that GST–Ssu72 downregulated significantly Th17 cell differentiation (Fig. 8A). Concentrations of IL-17 and TNF-α were decreased significantly in culture supernatants from the cells treated with GST–Ssu72 compared with those from GST treated the cells (Fig. 8B). These results demonstrated that Ssu72 can decrease Th17 cell differentiation and inflammatory response through inhibition of STAT3 activation *in vitro*.

## Discussion

Although the phosphatase function of Ssu72 has been investigated extensively^13, 14^, researchers have not yet determined whether it dephosphorylates STAT3. Additionally, little is known regarding the activity of Ssu72 in T cell-mediated immune responses and inflammatory autoimmune diseases. Here, we established for the first time the therapeutic activity of Ssu72 in RA and discovered an anti-inflammatory pathway by which it attenuates RA related to STAT3 activation both *in vitro* and *in vivo*.

The most notable observation of the present study is that Ssu72 improved CIA by reducing STAT3 activation. To the best of our knowledge, this study is the first to provide evidence indicating that Ssu72 may be an effective therapeutic molecule for the treatment of RA. The inhibition of STAT3 activation has been suggested to exacerbate the development of experimental autoimmune arthritis^7, 21^. Additionally, STAT3 activation increases the number of IL-17-producing Th17 cells and exacerbates the development of RA^22, 23^. Based on our findings, Ssu72 attenuated STAT3 activation and Th17 cell differentiation, crucial activities that are well known to ameliorate the progression of RA, indicating a novel putative therapeutic approach to regulate RA by modulating Ssu72 expression.

RA is characterized by excessive inflammation, erosive polyarthritis that is suggestive of osteoclastogenesis in the synovium, and the presence of serum autoantibodies^24, 25^. In the present study, osteoclastogenesis, total serum IgG levels, and the mRNA levels of the proinflammatory cytokines receptor activator of nuclear factor κ-B ligand (*RANKL*) and *TRAP* were significantly downregulated in arthritic joints from the Ssu72-overexpressing group compared with the mock group. An imbalance between Th17 and Treg cells contributes to the severity of RA^26^, and treatments modulating the reciprocal balance between these cell types play important roles in RA therapy^27^. TBK1 also represents a potential therapeutic target in RA^28^. Ssu72 ameliorated CIA progression by suppressing the inflammatory response and altering of the reciprocal balance between Th17 and Treg cells *in vivo*.

The levels of proinflammatory cytokines, including IL-1β, IL-17, IL-21, and TNF-α, are involved in the pathogenesis of RA^29^. Moreover, the levels of Th1 cytokines, such as interferon-γ (IFN-γ), IL-1β, and TNF-α, which mediate rheumatoid inflammation, are elevated in patients with RA^30, 31^. In contrast to Th1 cytokines, Th2 cytokines, such as IL-4, are expressed at low levels in RA joints and have a therapeutic effect on CIA^32, 33^. Th1 cell differentiation was significantly reduced in the spleens of mice in the Ssu72-overexpressing group compared with that in the mock group; however, Th2 cell differentiation was not significantly different between groups (Supplementary Figure 7A). *Ssu72* overexpression significantly increased Th2 differentiation in the lymph nodes of the treated group compared with that in the mock control group, but Th1 cell differentiation was not reduced (Supplementary Figure 7B). Considering the roles of Th17 cells and IL-17 in causing excess inflammation during the development of RA, our results suggest that Ssu72 may represent a novel therapeutic modality for regulating the differentiation of Th1 or Th2 cells in the treatment of RA.

We analyzed the relative *Ssu72* mRNA levels in peripheral blood mononuclear cells and CD4^+^ T cells from both healthy individuals and patients with RA using the National Center for Biotechnology Information Gene Expression Omnibus database (data sets GSE15573 and GSE4588). In our analysis, the levels of *Ssu72* mRNA were lower in patients with RA than in healthy controls (Supplementary Figure 8A and B). We also analyzed the degree of methylation of *Ssu72* in peripheral blood leukocytes using this database and found that methylation was significantly increased in cells from patients with RA compared with that in healthy individuals (Supplementary Figure 8C). The GSE42861 database includes 689 normal controls and 681 patients with RA, along with both clinical and pathological data. Hypermethylation of *Ssu72* might reduce Ssu72 expression and aggravate the progression of RA. In this study, Ssu72 overexpression attenuated the severity of CIA in a mouse model. Thus, upregulation of Ssu72 expression may represent a therapeutic strategy for the treatment of RA.

As phosphatase activity has been implicated in the pathogenesis of RA, approaches that modulate the activity of phosphatases can be used to treat RA. Based on emerging evidence, the regulation of phosphatase activity may improve outcomes in experimental models of arthritis, such as the CIA and K/BxN serum transfer mouse models^34, 35^. Moreover, overexpression of phosphatases such as PTEN and DUSP5, which inhibit STAT3 activation, revealed therapeutic activity and the attenuation of CIA development by downregulating Th17 cell differentiation^36, 37^. In this study, Ssu72 suppressed STAT3 activation and CIA progression by reducing Th17 cell differentiation. Thus, Ssu72 may have therapeutic potential to improve RA.

Previously, the expression levels of the *Ssu72* gene had been measured in fibroblast-like synoviocytes from patients with RA^38^. Diminished Ssu72 expression may be associated with the development of RA; however, limited data are available regarding the potential anti-arthritic effects of Ssu72. Our findings provide the first evidence showing that Ssu72 overexpression may attenuate autoimmune disease in an animal model. The therapeutic functions of Ssu72 identified here indicate that Ssu72 may substantially ameliorate RA progression by suppressing STAT3 activation and downregulating Th17 differentiation. Based on these preliminary results, Ssu72 may represent a strong candidate to target in the treatment of RA.

## Materials and Methods

### Animals

Six- to eight-week old male DBA1/J mice (SLC, Inc., Shizuoka, Japan) were maintained in cohorts of five mice in polycarbonate cages in a specific pathogen-free environment and were fed standard mouse chow (Ralston Purina, Gray Summit, MO, USA) and water ad libitum. All experimental procedures were examined and approved by the Animal Research Ethics Committee of The Catholic University of Korea.

### Ethics statement

The Animal Care Committee of The Catholic University of Korea approved the experimental protocol. All experimental procedures were evaluated and performed in accordance with protocols approved by the Animal Research Ethics Committee at The Catholic University of Korea (permit number: CUMC- 2016-0073-01). All procedures performed in the study followed ethical guidelines for animal studies.

### CII immunization and induction of CIA

CIA was induced in DBA1/J mice (n = 10 per group). Mice were intradermally immunized in the base of the tail with 100 μg of chicken CII (Chondrex Inc., Redmond, WA, USA) in either complete or incomplete Freund’s adjuvant (Chondrex Inc.).

### Clinical scoring and histological assessment of arthritis

One week after the CII immunization, mice with CIA were intravenously injected with 100 μg of the Ssu72 overexpression vector in 2 ml of saline over a 10-s period. After 2 weeks, the same mice received an intramuscular injection of 50 μg of the Ssu72 overexpression vector in the leg using electrical stimulation (i.e., electroporation). Mice were examined visually twice per week for the appearance of arthritis in the peripheral joints, which was graded using a previously reported index^39^. The final value represents the average index from all four legs, as recorded by two independent observers. For histological assessments, the joints of each mouse were fixed in 10% formalin, decalcified in 10% EDTA, and embedded in paraffin wax. Hematoxylin and eosin-stained sections were scored for inflammation, pannus invasion, and bone and cartilage damage. Scores were measured using published criteria^40^.

### Real-time polymerase chain reaction (PCR)

Total RNA was extracted using TRI Reagent (Molecular Research Center, Inc. Cincinnati, OH, USA) according to the manufacturer’s protocol. Complementary DNA was synthesized using a Super Script Reverse Transcription system (Takara). A Light-Cycler 2.0 instrument (software version 4.0; Roche Diagnostics) was used for PCR amplification. All reactions were conducted using Light Cycler Fast Start DNA Master SYBR Green I mix (Takara) according to the manufacturer’s instructions. Primer sequences used to amplify mouse genes are listed in Supplementary Table 1. All mRNA levels were normalized to the levels of the β-actin mRNA.

### Flow cytometry

Cells were stained with various combinations of fluorescent antibodies against CD4, CD25, FOXP3, IFN-γ, IL-4, and IL-17 (eBioscience, San Diego, CA, USA). Prior to intracellular staining, cells were restimulated for 4 h with phorbol myristate acetate (25 ng/ml) and ionomycin (250 ng/ml) in the presence of GolgiStop (BD Biosciences). Intracellular staining was performed using a kit (eBioscience) according to the manufacturer’s instructions. Flow cytometry was conducted on a FACSCalibur flow cytometer (BD Biosciences).

### Measurements of the IgG levels

Blood was obtained from the orbital sinuses of the mice, and serum was stored at −20 °C until further use. Serum IgG levels were examined using a commercially available ELISA kit (Bethyl Laboratories, Montgomery, TX, USA). Horseradish peroxidase (HRP) activity was measured using tetramethylbenzidine (eBioscience, San Diego, CA, USA). The absorbance was measured at 450 nm.

### Confocal microscopy and immunostaining

Spleen tissues were obtained 67 days after CII immunization, snap-frozen in liquid nitrogen, and stored at −80 °C. Tissue cryosections (7-μm thick) were fixed with acetone and stained with FITC-, PE-, PerCP-Cy5.5-, or allophycocyanin-conjugated monoclonal antibodies against mouse CD4, p-STAT3 (Tyr705, Ser727), p-STAT5, IL-17, and FOXP3 (eBioscience). After an overnight incubation at 4 °C, stained sections were visualized using confocal microscopy (LSM 510 Meta; Zeiss, Göttingen, Germany).

### Immunohistochemistry

Immunohistochemistry was performed using a Vectastain ABC kit (Vector Laboratories). Tissues were first incubated with primary antibodies against IL-1β, IL-6, IL-17, IL-21, TNF-α or RANKL overnight at 4 °C, probed with a biotinylated secondary antibody, and then stained with a streptavidin-peroxidase complex for 1 h. The final color product was developed using 3,3′-diaminobenzidine as the chromogen (Dako).

### TRAP staining

Decalcified ankle joints were processed for paraffin embedding, from which 7-μm-thick tissue sections were prepared. Sections were stained for TRAP using a Leukocyte Acid Phosphatase kit (Sigma–Aldrich, St. Louis, MO, USA) according to the manufacturer’s protocol.

### Transfection

The *Ssu72* overexpression vector was obtained from Professor Chang-Woo Lee (Department of Molecular Cell Biology, Sungkyunkwan University School of Medicine, Suwon, Gyeonggi, Korea) and used to overexpress Ssu72^41^. Mock or Ssu72 overexpression vector constructs were transfected into NIH-3T3 cells using X-tremeGENE HP Reagent (Roche) according to the manufacturer’s recommendations.

### Dual-luciferase assay

The construct containing the *Il17a* promoter firefly luciferase reporter (plasmid #20128) was kindly provided by Professor Chen Dong (Department of Immunology, M.D. Anderson Cancer Center, Houston, TX, USA) and used in the dual-luciferase assay. NIH-3T3 cells were transfected with the luciferase reporter construct or a control *Renilla* luciferase reporter plasmid using X-tremeGENE HP reagent (Roche, Mannheim, Germany) according to the manufacturer’s instructions. The dual-luciferase reporter system (Promega, Madison, WI, USA) was used to measure firefly and *Renilla* luciferase activities. Transfection efficiency and luciferase activity were both normalized to *Renilla* luciferase.

### RNA interference and mutagenesis

An siRNA construct targeting the *Ssu72* mRNA and a non-targeting siRNA (Santa Cruz Biotechnology, Santa Cruz, CA, USA) were used with the transfection system to knockdown *Ssu72* expression. Cells were transfected with 100 nM siRNA and X-tremeGENE HP reagent (Roche, Mannheim, Germany) according to the manufacturer’s recommendations.

Wild-type FLAG-tagged estrogen related receptor alpha (ESRRA) and HA-tagged histone deacetylase 4 (HDAC4) were subcloned into pcDNA3.3 (K8300-01; Invitrogen), point mutations corresponding to analogous human mutations were introduced into these sequences using the GeneArt Site-Directed Mutagenesis System (A13282; Invitrogen).

### CD4^+^ T cell isolation and differentiation into Th17

Splenocytes were harvested in ACK lysis buffer, washed, and resuspended in complete culture medium (RPMI 1640 supplemented with 10% [v/v] heat-inactivated fetal calf serum). To purify CD4^+^ T cells, mice splenocytes were incubated with CD4-coated magnetic beads and isolated on MACS columns for cell separation (Miltenyi Biotec, San Diego, CA, USA). CD4^+^ T cells were stimulated with plate-bound anti-CD3 mAb at 0.5 μg/ml and anti-CD28 mAb at 1 μg/ml (BD PharMingen, CA, USA) for 3 days using either Th17 cell-polarizing conditions (anti-IFN-γ at 2 μg/ml, anti-IL-4 at 2 μg/ml, TGF-β at 2 ng/ml, and IL-6 at 20 ng/ml).

### Purification of the recombinant GST-Ssu72 protein

Full-length human Ssu72 was cloned into a pGEX-KG plasmid vector. GST-Ssu72 and cell-penetrating peptides (CPPs; G-RKKRRQRRR-G) were subcloned into His-tagged fusion plasmids to generate recombinant His-CPP-fused GST-Ssu72 to improve the delivery of the recombinant protein into cells. The recombinant His-CPP-fused GST-Ssu72 protein was expressed in *Escherichia coli* BL21 and the resulting pellet was resuspended in lysis buffer [sodium phosphate (50 mmol/l, pH 8.0), NaCl (300 mmol/l), Triton X-100 (1%), imidazole (20 mmol/l), glycerol (10%), and phenylmethyl sulfonyl fluoride (PMSF; 1 mmol/l)] containing a protease inhibitor cocktail followed by sonication. Lysates were centrifuged at 20,000 × *g* for 20 min at 4 °C, and the supernatants were incubated with Ni-NTA agarose resin (Qiagen, 30230) and passed through a chromatography column (Bio-Rad, 731–1550). The resin was washed three times with wash buffer containing 20 mmol/l imidazole, and recombinant protein was eluted using elution buffer containing 250 mmol/l imidazole.

### Western blot analysis

Total proteins were extracted with lysis buffer containing 1% Nonidet P-40, PMSF, 2 mM sodium vanadate, 0.1% sodium deoxycholate, and a protease inhibitor mixture (Roche Applied Science, Mannheim, Germany). Proteins were loaded onto 10% polyacrylamide gels, subjected to SDS-PAGE, and the bands were then transferred to nitrocellulose membranes (Invitrogen Life Technologies, Carlsbad, CA, USA). Membranes were blocked with 5% (w/v) nonfat milk in Tris-buffered saline containing 0.1% Tween-20 for 1 h at room temperature and incubated with antibodies against p-STAT3 Y705, p-STAT3 S727, total STAT3 (Cell Signaling Technologies), Ssu72, β-actin (Santa Cruz Biotechnology), or FLAG (Sigma-Aldrich) overnight at 4 °C. Membranes were then incubated with HRP-conjugated goat anti-mouse or goat anti-rabbit antibodies. Immunoreactivity was determined using enhanced chemiluminescence reagents (Amersham Biosciences, Piscataway, NJ, USA).

### Statistical analysis

Statistical analyses were conducted using the nonparametric Mann-Whitney *U*-test for comparisons between two groups or one-way ANOVA with Bonferroni’s post hoc test for multiple comparisons. GraphPad Prism (ver. 5.01) was used for all analyses. *P* < 0.05 was used as the threshold for statistical significance. The data are presented as the mean ± standard deviation (SD).

## Electronic supplementary material

Supplementary Information
