# Supplementary material for: Ssu72 attenuates autoimmune arthritis via targeting of STAT3 signaling and Th17 activation
Source: Sci Rep. 2017 Jul 14;7:5506. doi: 10.1038/s41598-017-05421-x (PMC5511296; doi:10.1038/s41598-017-05421-x)
Supplement: Supplementary file 1 — Supplementary Information [file 41598_2017_5421_MOESM1_ESM.pdf]

Ssu72 attenuates autoimmune arthritis via targeting of STAT3 signaling and Th17 activation

Subtitle: Ssu72 attenuates CIA via targeting of STAT3

Seung Hoon Lee[1], Eun-Kyung Kim[1], Jeong-Eun Kwon[1], Jin-Kwan Lee[2], DoHyeong Lee[3], Se-Young Kim[1], Hyeon-Beom Seo[1], Hyun Sik Na[1], KyoungAh Jung[4], Seung-Ki Kwok[5], Chang-Woo Lee[2, 3], Sung-Hwan Park[1, 5 *] and Mi-La Cho[1, 6 *]

[1] The Rheumatism Research Center, Catholic Research Institute of Medical Science, The Catholic University of Korea, Seoul, South Korea

[2] Department of Health Sciences and Technology, SAIHST, Sungkyunkwan University, Seoul 06351, Korea

[3] Department of Molecular Cell Biology, Sungkyunkwan University School of Medicine, Suwon 16419, Korea

[4] Impact Biotech, Seoul, 137-040, South Korea

[5] Center for Rheumatic Disease, Division of Rheumatology, Department of Internal Medicine, Seoul St. Mary's Hospital, College of Medicine, The Catholic University of Korea, Seoul, 137-701, South Korea

[6] Laboratory of Immune Network, Conversant Research Consortium in Immunologic disease, College of Medicine, The Catholic University of Korea

[*] Sung-Hwan Park and Mi-La Cho contributed equally to this work.

Correspondence:

Sung-Hwan Park, MD, PhD, Rheumatism Research Center, Catholic Institutes of Medical Science, The Catholic University of Korea, 505 Banpo-dong, Seocho-gu, Seoul 137-040, Korea (South), Phone: 82-2-2258-7467; Fax: 82-2-599-4287; E-mail: rapark@catholic.ac.kr

Mi-La Cho, PhD, Rheumatism Research Center, Catholic Institutes of Medical Science, The Catholic University of Korea, 505 Banpo-dong, Seocho-gu, Seoul 137-040, Korea (South), Phone: 82-2-2258-7467; Fax: 82-2-599-4287; E-mail: iammila@catholic.ac.kr

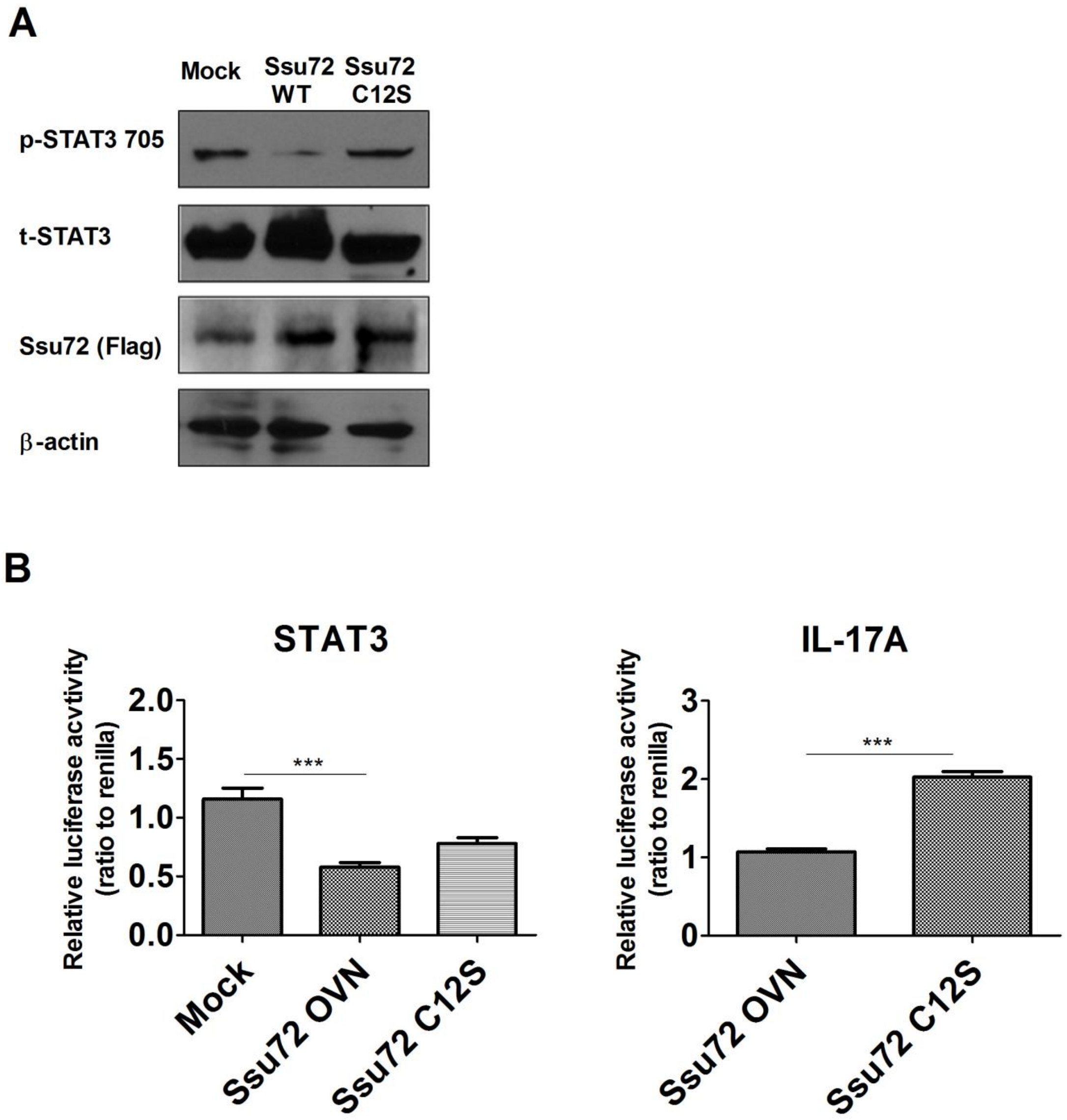

Supplementary figure 1. Ssu72 regulates the activation of STAT3. (A) The expression of p-STAT3 Tyr705 and p-STAT3 Ser727in NIH3T3 cells transfected with the mock, *Ssu72*wild-type-, or*Ssu72*(C12S)-expressing vector after stimulation by IL-6 (20ng/ml) for 1hwas determined by western blot analysis. (B) NIH3T3cells were transfected with the STAT3-regulating promoter and *Il17a*promoter luciferase vector. Luciferase activity was measured using a dual-luciferase reporter assay system. Data are presented as the mean ± SD of three independent experiments (*P < 0.05).

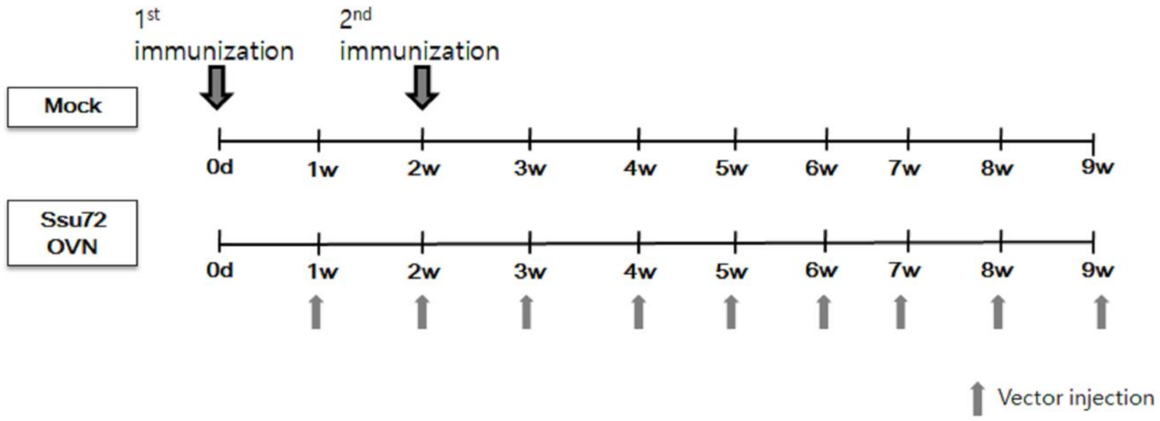

Supplementary figure 2. Schedule of CIA experiment using Ssu82 overexpression vector. CIA was induced as described in the Methods section. Mice were sacrificed on 9 weeks after first immunization. Schedule of vector injection into CIA mice.

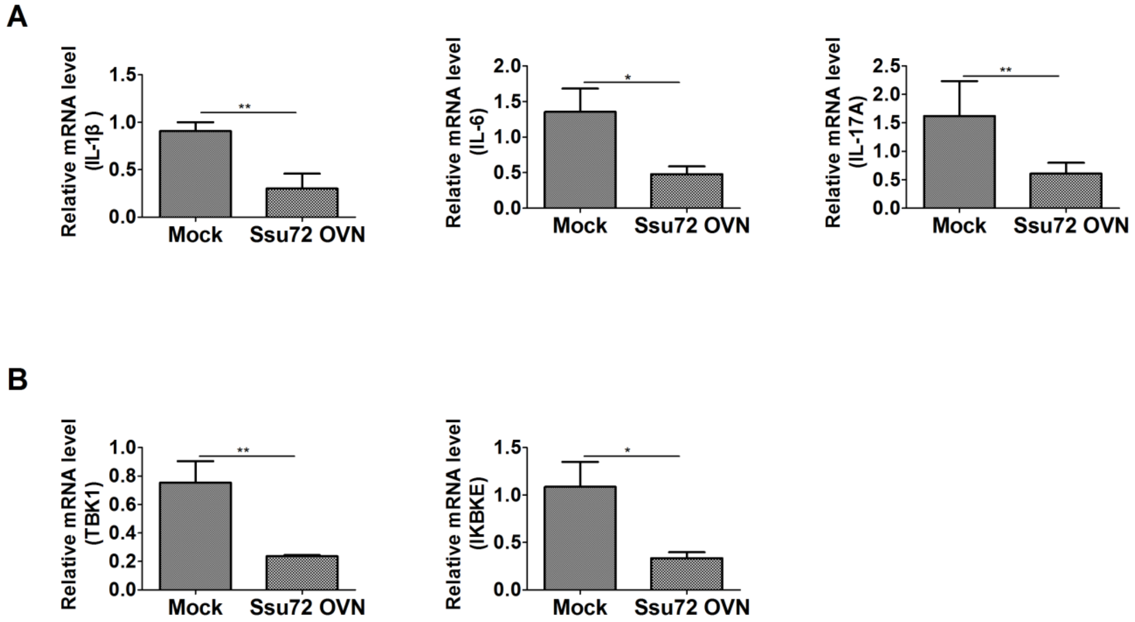

Supplementary figure 3. Ssu72 reduces inflammatory mediators. (A and B) Gene expression of IL-1β, IL-17A, IL-21, TBK1, and IKBKE in splenocytes from CIA mice was measured by real-time PCR.

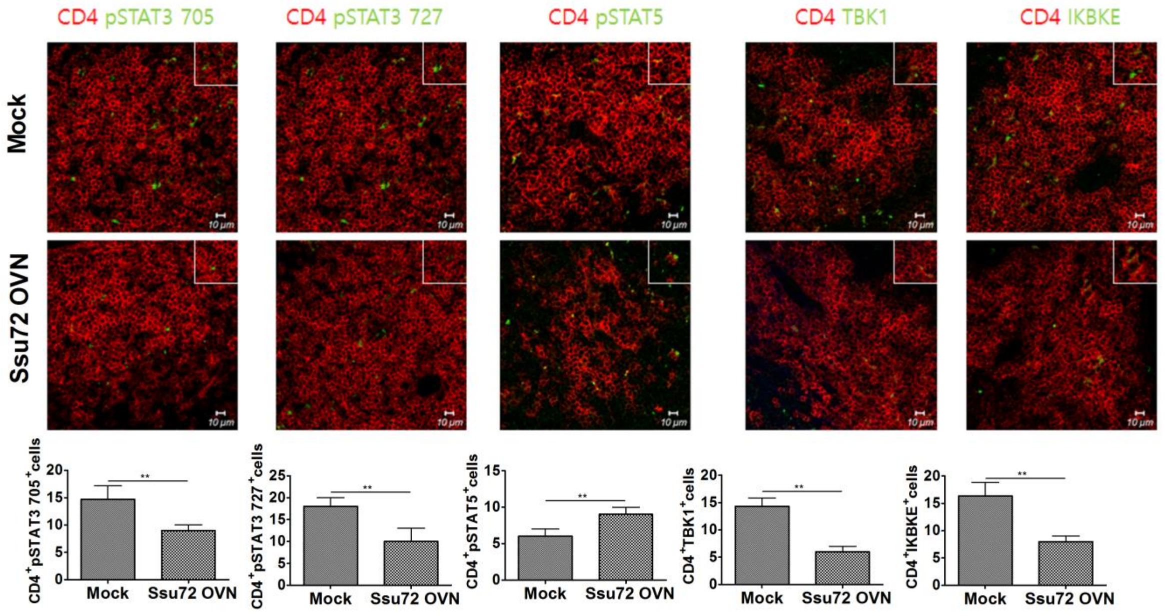

Supplementary figure 4. Ssu72 decreases STAT3 activation in CIA. (A) Spleens of CIA mice were subjected to immunostaining for $CD4^+$p-STAT3 $Tyr705^+$, $CD4^+$ p-STAT3 $Ser727^+$, $CD4^+$p-$STAT5^+$, $CD4^+TBK1^+$ and- $CD4^+IKBKE^+$ cells. (Scale bar, 10μm) The number of cells was counted in four independent quadrants. Data are presented as the mean ± SD of three independent experiments (*P < 0.05, ***P < 0.01, n = 6).

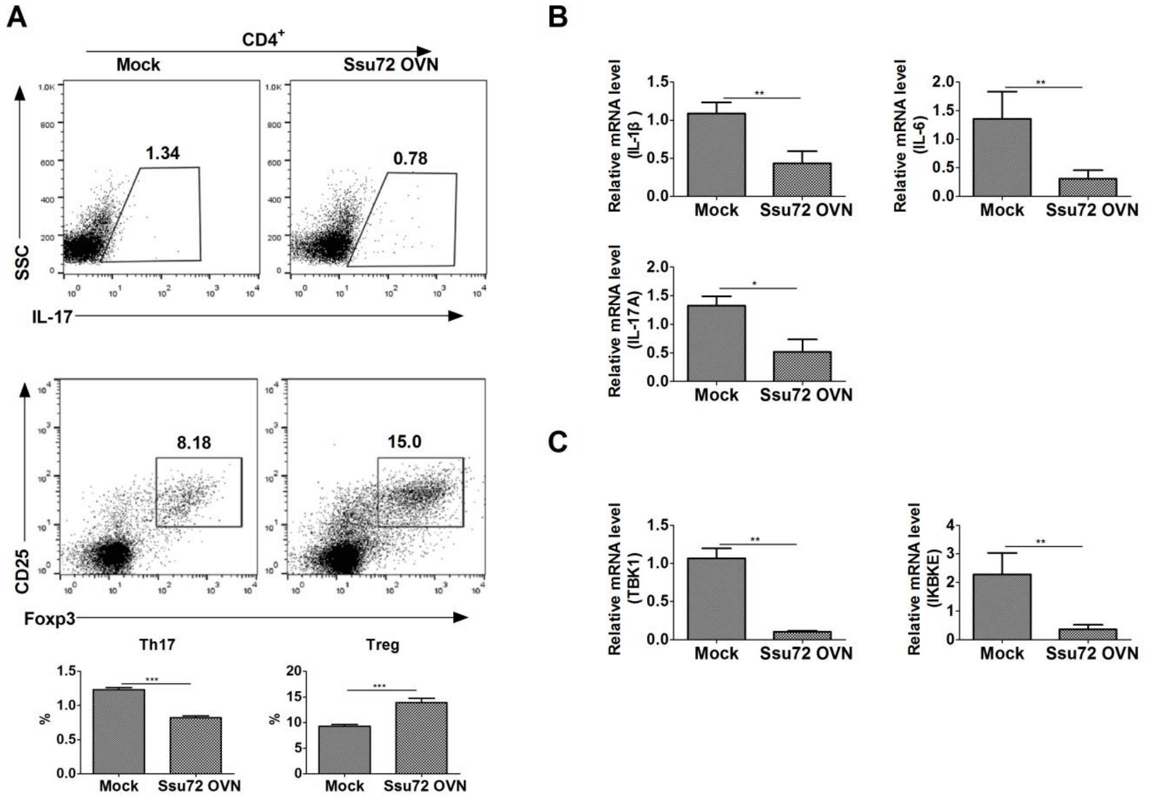

Supplementary figure 5. Ssu72 regulates reciprocal Th17/Treg balance in lymph node from CIA mice. (A) The populations of IL-17, CD25 and Foxp3 producing $CD4^+$ T cells in lymph node from CIA mice were analyzed by intracellular flow cytometric analysis. (B and C) Gene expression of IL-1β, -17A, -21, TBK1 and IKBKE in lymph node cells from CIA mice was measured by real-time PCR. Data are presented as the mean ± SD of three independent experiments (*P < 0.05, ***P < 0.01, n = 6).

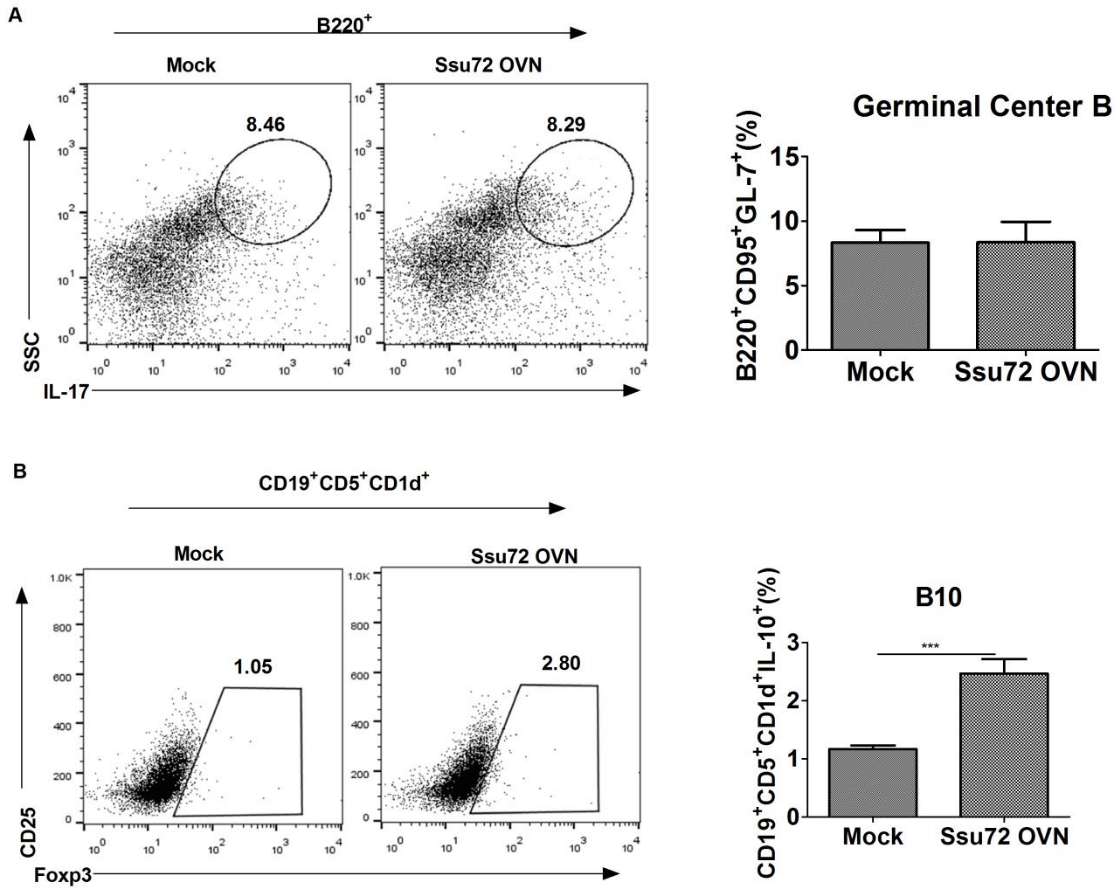

Supplementary figure 6. Ssu72 overexpression reveals therapeutic effect in CIA *in vivo* model controlling B cell response. (A) The populations of GL-7 producing B220^{+}CD95^{+} B cells in lymph node from CIA mice were analyzed by intracellular flow cytometric analysis. **(B**) The populations of IL-10 producing CD19^{+}CD5^{+}CD1d^{+} B cells in lymph node from CIA mice were analyzed by intracellular flow cytometric analysis. Data are presented as the mean ± SD of three independent experiments (*P < 0.05, ***P < 0.01, n = 6).

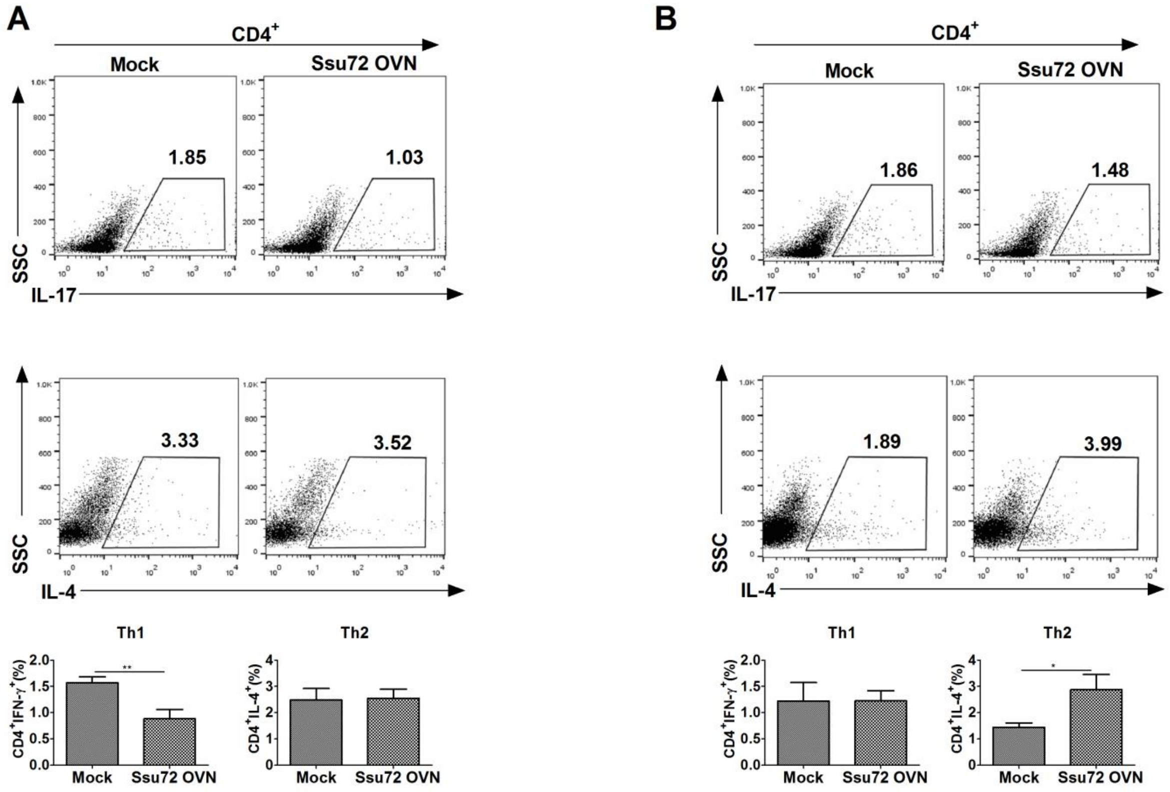

Supplementary figure 7. Ssu72 regulates reciprocal Th1/Th2 balance in CIA mice. (A and B) The populations of IFN-γ and IL-4 producing CD4$^+$ T cells in splenocytes and lymph node from CIA mice were analyzed by intracellular flow cytometric analysis. Data are presented as the mean ± SD of three independent experiments (*P < 0.05, ***P < 0.01, n = 6).

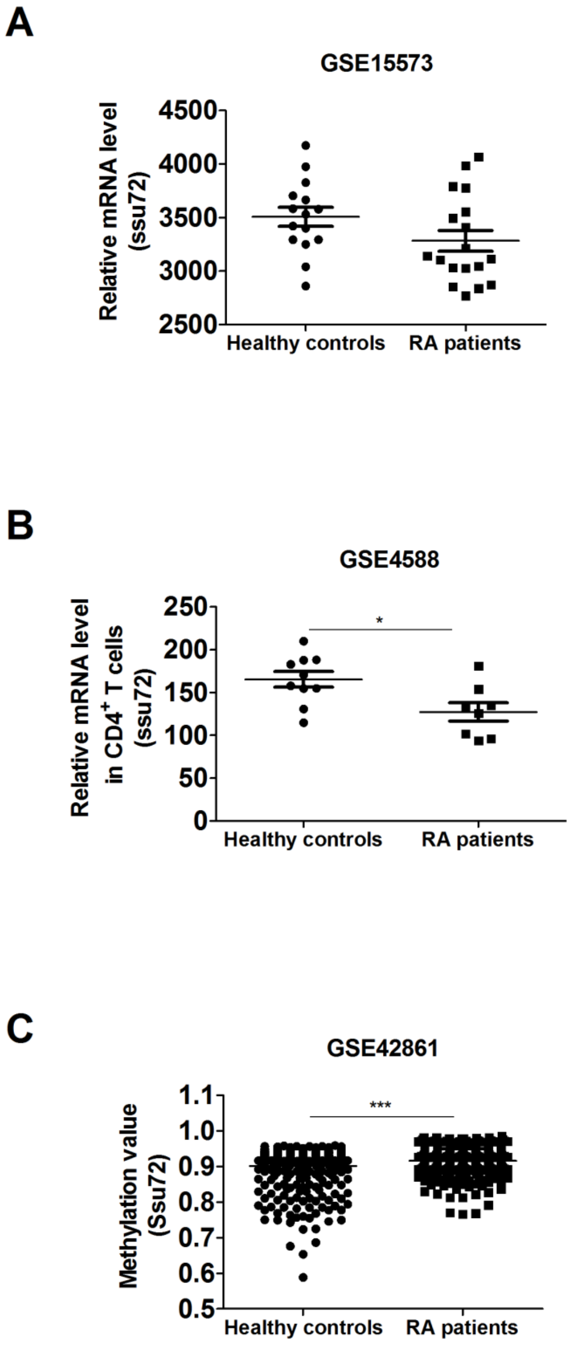

Supplementary figure 8. Ssu72 is related with RA patients. (A) Gene expression of Ssu72 in peripheral blood mononuclear cells of healthy individual and RA patients. (B) $CD4^+$ T cells sorted from peripheral blood mononuclear cells of healthy individual and RA patients. (C) Methylation value of Ssu72 in peripheral blood leukocytes of normal individual and RA patients. Data are presented as the mean ± SD (*P < 0.05, **P < 0.03).

Gene	Sense primer (5'-->3)	Antisense primer (3'-->5')
IL-17A	CCT CAA AGC TCA GCG TGT CC	GAG CTC ACT TTT GCG CCA AG
IL-1β	GGA TGA GGA CAT GAG CAC ATT C	GGA AGA CAG GCT TGT GCT CTG A
IL-6	AAC GAT GAT GCA CTT GCA GAA A	TCT GAA GGA CTC TGG CTT TGT C
IL-10	GGC CCA GAA ATC AAG GAG CA	AGA AAT CGA TGA CAG CGC CT
IL-6 receptor	ATT TGT GTG CTG AAG GAG GC	AAA GGA CAG GAT GTT GCA GG
IL-4	GGT TCT CAA CCC CCA GCT AGT	GCC GAT GAT CTC TCT CAA GT
TBK1	GAC ATG CCT CTC TCC TGT AGT C	GGT GAA GCA CAT CAC TGG TCT C
IKBKE	CCC AAA GTT CGT CCC TAA GGT TG	ATC AAC GCC TGT CCA TCC AGC A
NDUFB5	TCC CAG AAG GCT ACA TCC CT	ATT CCG GGC GAT CCA TCT TG
β-actin	GAAATCGTGCGTGACATCAAAG	TGTAGTTTCATGGATGCCACAG

Supplementary Table 1. PCR primer sequence used in this study.
